# Supplementary material for: Structured follow-up pathway to address unmet needs after transient ischaemic attack and minor stroke (SUPPORT TIA): Feasibility study and process evaluation
Source: PLoS One. 2025 Mar 13;20(3):e0317425. doi: 10.1371/journal.pone.0317425 (PMC11906085; doi:10.1371/journal.pone.0317425)
Supplement: S2 File — (DOCX) [file pone.0317425.s002.docx]

# Supporting information

**S1 Table: Progression criteria**

| **Key uncertainties** | **Measures used** | **Progression criteria** |
| --- | --- | --- |
| **Trial design** | | |
| Recruitment | % target sample size recruited | ● ≥90%: proceed to a full-scale trial |
|  |  | ● 70-89%: SOC will consider the feasibility of proceeding to a full-scale trial bearing in mind the data presented, representativeness of the sample, and possible steps to increase recruitment |
|  |  | ● <70%: full-scale trial unlikely to be feasible |
| Randomisation* | % of consented participants randomised | ● ≥90%: proceed to a full-scale trial |
|  |  | ● 70-89%: SOC will consider the feasibility of proceeding to a full-scale trial bearing in mind the data presented, representativeness of the sample, and possible steps to address randomisation issues |
|  |  | ● <70%: full-scale trial unlikely to be feasible |
| Return rate of 24-week questionnaire* | % of 24-week questionnaires returned | ● ≥80%: proceed to a full-scale trial |
|  |  | ● 50-79%: SOC will consider the feasibility of proceeding to a full-scale trial bearing in mind the data presented, representativeness of the sample, and possible steps to increase return rates |
|  |  | ● <50%: full-scale trial unlikely to be feasible |
| **Intervention** | | |
| Attendance rate* | % of intervention arm participants attending first appointment | ● ≥90%: proceed to a full-scale trial |
|  |  | ● 70-89%: SOC will consider the feasibility of proceeding to a full-scale trial bearing in mind the data presented, representativeness of the sample, and possible steps to increase attendance |
|  |  | ● <70%: full-scale trial unlikely to be feasible |
| Delivery of the intervention | % completion of: checklists, action plans, GP letters; use of directory of support services; Issues regarding delivery of the intervention components and contamination explored in qualitative interviews | The SOC will consider the quantitative and qualitative data and make an overall judgement on whether the intervention content is delivered as intended |
| Acceptability | % of participants reporting acceptability of intervention components on intervention feedback questionnaire; Issues regarding acceptability of the intervention components explored in qualitative interviews | The SOC will consider the quantitative and qualitative data and make an overall judgement on whether the intervention is acceptable |
| * critical progression criteria: the trial is unlikely to be feasible if these criteria are not met, even if other criteria are satisfactory.  GP: General Practitioner; SOC: Study Oversite Committee; %: percent | | |

**S2 Table: Summary of characteristics of participants for qualitative TIA/ minor stroke interviews, observations, GP letters and Action plans**

|  |  | **Qualitative interviews (n=11)** | **Observation (n=3)** | **GP letters/ Action plans (n=7)** |
| --- | --- | --- | --- | --- |
| **Sex** | **Male** | **3** | **3** | **1** |
|  | **Female** | **8** | **0** | **6** |
| **Site** | **Site 1** | **3** | **0** | **2** |
|  | **Site 2** | **7** | **3** | **5** |
|  | **Site 3** | **1** | **0** | **0** |
| **Ethnicity** | **White** | **11** | **3** | **7** |
| **Age** | **50-59** | **2** | **0** | **0** |
|  | **60-69** | **2** | **1** | **0** |
|  | **70-79** | **4** | **1** | **4** |
|  | **80-89** | **3** | **1** | **8** |
|  | **≥90** | **0** | **0** | **1** |
| **Diagnosis** | **TIA** | **9** | **2** | **7** |
|  | **Minor stroke** | **2** | **1** | **0** |
| **Employment** | **Retired** | **8** | **2** | **7** |
|  | **Unemployed** | **1** | **0** | **0** |
|  | **Employed** | **2** | **1** | **0** |
| **Randomisation** | **Intervention** | **8** | **3** | **7** |
|  | **Control** | **3** | **0** | **0** |

**S3 Table: Summary of characteristics of site staff qualitative participants**

| **ID** | **Site** | **Sex** | **Clinical role** | **Role in study** |
| --- | --- | --- | --- | --- |
| N1 | Site 1 | Female | Nurse | Recruitment |
| N2 | Site 2 | Female | Nurse | Recruitment, delivered intervention |
| N3 | Site 2 | Female | Nurse | Recruitment, delivered intervention |
| N4 | Site 3 | Male | Nurse | Recruitment, delivered intervention |

**S4 Table: Number of patients screened, eligible and recruited from each site**

|  |  | **Site 1** |  |  | **Site 2** |  |  | **Site 3** |  |
| --- | --- | --- | --- | --- | --- | --- | --- | --- | --- |
|  | **Screened** | **Eligible** | **Recruited** | **Screened** | **Eligible** | **Recruited** | **Screened** | **Eligible** | **Recruited** |
| **Sep-21** | 19 | 4 | 1 | - | - | - | - | - | - |
| **Oct-21** | 34 | 9 | 3 | - | - | - | - | - | - |
| **Nov-21** | 26 | 6 | 1 | 13 | 1 | 1 | - | - | - |
| **Dec-21** | 33 | 10 | 3 | 46 | 11 | 3 | - | - | - |
| **Jan-22** | 35 | 8 | 2 | 77 | 9 | 2 | - | - | - |
| **Feb-22** | 33 | 3 | 1 | 45 | 21 | 1 | - | - | - |
| **Mar-22** | 31 | 6 | 1 | 48 | 15 | 5 | - | - | - |
| **Apr-22** | 23 | 4 | 0 | 71 | 18 | 4 | NR | NR | 2 |
| **May-22** | 30 | 5 | 1 | 53 | 11 | 4 | NR | NR | 5 |
| **Jun-22** | 27 | 4 | 0 | 20 | 9 | 2 | NR | NR | 2 |
| **Jul-22** | NR | NR | 0 | 23 | 19 | 4 | NR | NR | 1 |
| **Aug-22** | NR | NR | 2 | 7 | 4 | 1 | NR | NR | 2 |
| **Total** | 291 | 59 | 15 | 403 | 118 | 27 | 129 | NR | 12 |
| NR: Not reported; - : site not yet recruiting | | | | | | |  |  |  |

**S5 Table: Recruitment per month, per site**

|  | **Months site is open, N** | **Recruitment total, N** | **Recruitment per month** | | | | | | | | | | | |
| --- | --- | --- | --- | --- | --- | --- | --- | --- | --- | --- | --- | --- | --- | --- |
|  |  |  | **Sep 21** | **Oct 21** | **Nov 21** | **Dec 21** | **Jan 22** | **Feb 22** | **Mar 22** | **Apr 22** | **May 22** | **Jun 22** | **July 22** | **Aug 22** |
| **Site 1** | **12** | **15** | 1 | 3 | 1 | 3 | 2 | 1 | 1 | 0 | 1 | 0 | 0 | 2 |
| **Site 2** | **10** | **27** | - | - | 1 | 3 | 2 | 1 | 5 | 4 | 4 | 2 | 4 | 1 |
| **Site 3** | **5** | **12** | - | - | - | - | - | - | - | 2 | 5 | 2 | 1 | 2 |
| **Total** | **12** | **54** | **1** | **3** | **2** | **6** | **4** | **2** | **6** | **6** | **10** | **4** | **5** | **5** |

**S6 Table: Method of consent, per site**

|  | **Face-to-face** | **Postal** | **Verbal** | **Total** |
| --- | --- | --- | --- | --- |
| **Site 1** | 7 | 7 | 1 | 15 |
| **Site 2** | 0 | 27 | 0 | 27 |
| **Site 3** | 12 | 0 | 0 | 12 |
| **Total** | 19 | 34 | 1 | 54 |

**S7 Table: Summary of follow-up questionnaire return rate**

| **Timepoint** | **Total, N (%)** | **Intervention, N (%)** | **Control, N (%)** |
| --- | --- | --- | --- |
| 1 week | 51 (94.4) | 24 (96.0) | 27 (93.1) |
| 12 weeks | 46 (85.2) | 20 (80.0) | 26 (89.7) |
| 24 weeks* | 27 (71.1) | 13 (65.0) | 14 (77.8) |

*38 participants eligible to complete the 24-week questionnaire (intervention n= 20; control n= 18)

**S8 Table: Data completeness of participant completed questionnaires: frequency of questionnaires returned complete**

|  | **Total, N (%)** | **Intervention, N (%)** | **Control, N (%)** |
| --- | --- | --- | --- |
| **Post-baseline** | **n=51** | **n=24** | **n=27** |
| **HADS** | 49 (96.1) | 24 (100.0) | 25 (92.6) |
| **EQ-5D** | 51 (100.0) | 24 (100.0) | 27 (100.0) |
| **EQ-5D VAS** | 51 (100.0) | 24 (100.0) | 27 (100.0) |
| **PROMIS-10** | 49 (96.1) | 24 (100.0) | 25 (92.6) |
| **FAS** | 49 (96.1) | 23 (95.8) | 26 (96.3) |
| **MARS-5** | 50 (98.0) | 23 (95.8) | 27 (100.0) |
| **PAM-13** | 48 (94.1) | 23 (95.8) | 25 (92.6) |
| **Satisfaction with care** | 51 (100.0) | 24 (100.0) | 27 (100.0) |
| **12 weeks** | **n=46** | **n=20** | **n=26** |
| **HADS** | 42 (91.3) | 20 (100.0) | 22 (84.6) |
| **EQ-5D** | 44 (95.7) | 19 (95.0) | 25 (96.2) |
| **EQ-5D VAS** | 44 (95.7) | 19 (95.0) | 25 (96.2) |
| **PROMIS-10** | 45 (97.8) | 20 (100.0) | 25 (96.2) |
| **FAS** | 44 (95.7) | 19 (95.0) | 25 (96.2) |
| **MARS-5** | 45 (97.8) | 20 (100.0) | 25 (96.2) |
| **PAM-13** | 38 (82.6) | 14 (70.0) | 24 (92.3) |
| **Satisfaction with care** | 44 (95.7) | 18 (90.0) | 26 (100.0) |
| **24 weeks** | **n=27** | **n=13** | **n=14** |
| **HADS** | 26 (96.3) | 12 (92.3) | 14 (100.0) |
| **EQ-5D** | 25 (92.6) | 12 (92.3) | 13 (92.9) |
| **EQ-5D VAS** | 26 (96.3) | 13 (100.0) | 13 (92.9) |
| **PROMIS-10** | 25 (92.6) | 13 (100.0) | 12 (85.7) |
| **FAS** | 25 (92.6) | 11 (84.6) | 14 (100.0) |
| **MARS-5** | 25 (92.6) | 11 (84.6) | 14 (100.0) |
| **PAM-13** | 25 (92.6) | 12 (92.3) | 13 (92.9) |
| **Satisfaction with care** | 25 (92.6) | 11 (84.6) | 14 (100.0) |
| EQ-5D-5L: EuroQol 5-Dimensions; FAS: Fatigue Assessment Scale; HADS: Hospital Anxiety and Depression Scale; MARS-5: Medication Adherence Rating Scale -5; PAM: Patient Activation Measure-13; PROMIS-10: Patient-Reported Outcomes Measurement Information System (PROMIS)- Global Health 10 | | | |

**S9 Table: Data completeness of health economic questionnaires: frequency of missing data**

|  | **12 weeks** | | | **24 weeks** | | |
| --- | --- | --- | --- | --- | --- | --- |
|  | **Total (n=46)** | **Intervention (n=20)** | **Control (n=26)** | **Total (n=27)** | **Intervention (n=13)** | **Control (n=14)** |
|  | **N (%)** | **N (%)** | **N (%)** | **N (%)** | **N (%)** | **N (%)** |
| **Employment** |  |  |  |  |  |  |
| **1. What is your employment status?** | 1 (2.2) | 0 (0.0) | 1 (3.85) | 0 (0.0) | 0 (0.0) | 0 (0.0) |
| **2. In the last 3 months has your employment status or working pattern changed due to your mini stroke (TIA) or minor stroke?** | 0 (0.0) | 0 (0.0) | 0 (0.0) | 0 (0.0) | 0 (0.0) | 0 (0.0) |
| **3. In the last 3 months have you taken any days off sick from work due to your mini stroke (TIA) or minor stroke?** | 0 (0.0) | 0 (0.0) | 0 (0.0) | 0 (0.0) | 0 (0.0) | 0 (0.0) |
| **4. In the last 3 months have your hours of work changed due to your mini stroke (TIA) or minor stroke?** | 0 (0.0) | 0 (0.0) | 0 (0.0) | 0 (0.0) | 0 (0.0) | 0 (0.0) |
| **Usual activities** |  |  |  |  |  |  |
| **5. Over the last 3 months, approximately how many days has your mini stroke (TIA) or minor stroke stopped you undertaking these activities?** |  |  |  |  |  |  |
| Education | 20 (43.5) | 11 (55.0) | 9 (34.6) | 7 (25.9) | 3 (23.1) | 4 (28.6) |
| Childcare/ care of a relative | 18 (39.1) | 10 (50.0) | 8 (30.8) | 6 (22.2) | 3 (23.1) | 3 (21.4) |
| Housework | 14 (30.4) | 6 (30.0) | 8 (30.8) | 4 (14.8) | 2 (15.4) | 2 (14.3) |
| Voluntary work | 21 (45.7) | 12 (60.0) | 9 (34.6) | 9 (33.3) | 5 (38.5) | 4 (28.6) |
| Other | 28 (60.9) | 13 (65.0) | 15 (57.7) | 16 (59.3) | 8 (61.5) | 8 (57.1) |
| **6. Over the last 3 months, has a relative or friend taken time off work to look after you?** | 6 (13.0) | 1 (5.0) | 5 (19.2) | 1 (3.7) | 0 (0.0) | 1 (7.1) |
| **7. Have you incurred any other costs because of your mini stroke (TIA) or minor stroke over the last 3 months?** | 5 (10.9) | 2 (10.0) | 3 (11.5) | 1 (3.7) | 1 (7.7) | 0 (0.0) |
| **Healthcare and support** |  |  |  |  |  |  |
| **8. Have you been admitted to and/or discharged from hospital in the last 3 months?** | 2 (4.3) | 1 (5.0) | 1 (3.9) | 0 (0.0) | 0 (0.0) | 0 (0.0) |
| **9. In the last 3 months have you visited or received treatment from any of the people/ services listed below?** |  |  |  |  |  |  |
| Stroke doctor | 4 (8.7) | 3 (15.0) | 1 (3.9) | 1 (3.7) | 1 (7.7) | 0 (0.0) |
| Stroke nurse | 8 (17.4) | 6 (30.0) | 2 (7.7) | 2 (7.4) | 2 (15.4) | 0 (0.0) |
| GP | 5 (10.9) | 4 (20.0) | 1 (3.9) | 3 (11.1) | 2 (15.4) | 1 (7.1) |
| Practice nurse | 5 (10.9) | 4 (20.0) | 1 (3.9) | 3 (11.1) | 3 (23.1) | 0 (0.0) |
| Health visitor | 8 (17.4) | 4 (20.0) | 4 (15.4) | 2 (7.4) | 0 (0.0) | 2 (14.3) |
| Physiotherapist | 7 (15.2) | 4 (20.0) | 3 (11.5) | 2 (7.4) | 2 (15.4) | 0 (0.0) |
| Occupational therapist | 7 (15.2) | 4 (20.0) | 3 (11.5) | 2 (7.4) | 2 (15.4) | 0 (0.0) |
| Speech and language therapist | 7 (15.2) | 4 (20.0) | 3 (11.5) | 3 (11.1) | 2 (15.4) | 1 (7.1) |
| Psychologist | 9 (19.6) | 4 (20.0) | 5 (19.2) | 2 (7.4) | 2 (15.4) | 0 (0.0) |
| Dietician | 8 (17.4) | 4 (20.0) | 4 (15.4) | 4 (14.8) | 3 (23.1) | 1 (7.1) |
| Stroke rehabilitation: hospital | 8 (17.4) | 4 (20.0) | 4 (15.4) | 2 (7.4) | 2 (15.4) | 0 (0.0) |
| Stroke rehabilitation: community | 7 (15.2) | 4 (20.0) | 3 (11.5) | 2 (7.4) | 2 (15.4) | 0 (0.0) |
| Other | 18 (39.1) | 8 (40.0) | 10 (38.5) | 5 (18.5) | 5 (38.5) | 0 (0.0) |
| **10. In the last 3 months have you accessed any support services or groups for any of the reasons listed below?** |  |  |  |  |  |  |
| Exercise | 4 (8.7) | 2 (10.0) | 2 (7.7) | 0 (0.0) | 0 (0.0) | 0 (0.0) |
| Diet | 6 (13.0) | 2 (10.0) | 4 (15.4) | 0 (0.0) | 0 (0.0) | 0 (0.0) |
| Stopping smoking | 6 (13.0) | 2 (10.0) | 4 (15.4) | 2 (7.4) | 1 (7.7) | 1 (7.1) |
| Alcohol or drug addiction | 6 (13.0) | 3 (15.0) | 3 (11.5) | 1 (3.7) | 1 (7.7) | 0 (0.0) |
| Other healthy lifestyle service | 6 (13.0) | 3 (15.0) | 3 (11.5) | 1 (3.7) | 1 (7.7) | 0 (0.0) |
| Anxiety/ Depression | 4 (8.7) | 2 (10.0) | 2 (7.7) | 0 (0.0) | 0 (0.0) | 0 (0.0) |
| Sleep | 4 (8.7) | 2 (10.0) | 2 (7.7) | 0 (0.0) | 0 (0.0) | 0 (0.0) |
| Memory or thinking | 5 (10.9) | 2 (10.0) | 3 (11.5) | 0 (0.0) | 0 (0.0) | 0 (0.0) |
| Speech or communication | 4 (8.7) | 2 (10.0) | 2 (7.7) | 0 (0.0) | 0 (0.0) | 0 (0.0) |
| Relationships with family or friends | 5 (10.9) | 3 (15.0) | 2 (7.7) | 0 (0.0) | 0 (0.0) | 0 (0.0) |
| Return to work | 5 (10.9) | 2 (10.0) | 3 (11.5) | 1 (3.7) | 0 (0.0) | 1 (7.1) |
| Charity | 4 (8.7) | 2 (10.0) | 2 (7.7) | 0 (0.0) | 0 (0.0) | 0 (0.0) |
| Support group | 6 (13.0) | 3 (15.0) | 3 (11.5) | 1 (3.7) | 1 (7.7) | 0 (0.0) |
| Other | 5 (10.9) | 3 (15.0) | 2 (7.7) | 1 (3.7) | 1 (7.7) | 0 (0.0) |

**S10 Table: Summary of intervention attendance rate, mode of delivery and length of appointment (N=24)**

|  |  | **Site 1** | **Site 2** | **Site 3** | **Total** |
| --- | --- | --- | --- | --- | --- |
| Randomised to intervention arm | N | 7 | 14 | 4 | 25 |
| Attended | N (%) | 7 (100.0) | 14 (100.0) | 3 (75.0) | 24 (96.0) |
| Mode of delivery | Telephone N (%) | 7 (100.0) | 14 (100.0) | 2 (66.7) | 23 (95.8) |
|  | Face-to-face N (%) | 0 (0.0) | 0 (0.0) | 1 (33.3) | 1 (4.2) |
| Length of appointment (minutes) | Median [IQR] | 30 [20, 40] | 20 [20, 30] | 25 [15, 25] | 22.5 [20, 30] |
|  | Range | 20-65 | 10-45 | 15-25 | 10-65 |

**S11 Table: Summary of checklist items checked (n=19).**

|  |  | N (%) |
| --- | --- | --- |
| **Information about** | Diagnosis | 7 (36.8) |
|  | Stroke risk | 12 (63.2) |
|  | Driving | 3 (15.8) |
| **Advice about** | Stroke prevention medication | 10 (52.6) |
|  | Medication side effects | 6 (31.6) |
|  | Lifestyle change | 9 (47.4) |
| **Fatigue** | I feel tired most of the time or I get easily tired | 13 (68.4) |
|  | I find it difficult to concentrate and do things | 6 (31.6) |
| **Mood** | I feel anxious | 6 (31.6) |
|  | I feel depressed | 5 (26.3) |
|  | I experience anger, frustration or mood swings | 2 (10.5) |
|  | I feel that my personality has changed | 1 (5.3) |
| **Memory and thinking** | I find it difficult to think, concentrate, or remember things | 4 (21.1) |
| **Communication** | I find it difficult to understand / communicate with others | 1 (5.3) |
|  | I have problems with speech, word finding or talking to others | 4 (21.1) |
| **Physical** | I experience muscle weakness or problems with balance | 3 (15.8) |
|  | I have headaches | 4 (21.1) |
|  | I am sensitive to noise or light | 4 (21.1) |
| **Incontinence** | I am having a problem controlling my bladder or bowels | 2 (10.5) |
| **Intimate relationships** | Since my mini stroke I have problems with sex | 2 (10.5) |
| **Work or education** | I am having problems at work or education | 0 (0.0) |
|  | I would like support and advice on returning to work or education | 1 (5.3) |
| **Relationships** | My personal relationships with my family or friends have become difficult or stressed | 3 (15.8) |
| **Social activities/ daily tasks** | I find it difficult to take part in hobbies or leisure activities | 1 (5.3) |
|  | I have difficulty doing daily tasks | 3 (15.8) |

**S12 Table: Observation checklists from 3 intervention observations**

|  | **Observation 1** | | | **Observation 2** | | | **Observation 3** | | |
| --- | --- | --- | --- | --- | --- | --- | --- | --- | --- |
|  | **Yes** | **No** | **N/A** | **Yes** | **No** | **N/A** | **Yes** | **No** | **N/A** |
| **Participant completed the checklist** | X |  |  | X |  |  | X |  |  |
| **Checklist used by HCP** | X |  |  | X |  |  | X |  |  |
| **Discussed needs** |  |  |  | X |  |  | X |  |  |
| **Blood pressure taken** |  |  | X |  |  | X |  |  | X |
| **Blood pressure actioned (if applicable)** |  |  | X |  |  | X |  |  | X |
| **Needs actioned/ actions discussed** | X |  |  | X |  |  | X |  |  |
| **Used local directory** |  |  | X |  |  | X |  | X |  |
| **Action plan developed** | X |  |  | X |  |  | X |  |  |
| **Action plan discussed and agreed with participant** | X |  |  | X |  |  | X |  |  |
| **Participant provided with website** |  | X |  |  |  | X |  | X |  |
| **Participant provided with brochure** | X |  |  | X |  |  | X |  |  |
| **Copy of action plan sent to participant** | X |  |  | X |  |  | X |  |  |
| **Copy of GP letter sent to participant** | X |  |  | X |  |  | X |  |  |
| **Letter sent to GP with action plan** | X |  |  | X |  |  | X |  |  |
| **GP letter template used?** | X |  |  | X |  |  | X |  |  |
| **Further follow up required?** |  | X |  |  | X |  |  | X |  |
| N/A: Not Applicable | | | | | | | | | |

**S13 Table: Recommended actions from the Action Plan**

| **Action** | **N (%)** |
| --- | --- |
| Read TIA/minor stroke booklet: specific sections highlighted | 11 (45.8) |
| Access specific services from local services directory | 10 (41.7) |
| Monitor Blood Pressure at home | 6 (25.0) |
| Read Stroke Association Fatigue leaflet | 6 (25.0) |
| No specific actions | 5 (20.8) |
| Read TIA/minor stroke booklet: general | 4 (16.7) |
| Read Anxiety Self Help Guide | 3 (12.5) |
| Use recommended App | 2 (8.3) |
| Specific lifestyle advice | 2 (8.3) |
| Contact GP for medication review | 2 (8.3) |
| Contact GP for emotional support | 1 (4.2) |
| Contact GP for BP check | 1 (4.2) |
| Stroke Association Relationships website | 1 (4.2) |

**S14 Table: GP recommendations from GP letters**

| **GP recommendation** | **N (%)** |
| --- | --- |
| No specific actions for GP | 10 (41.7) |
| Anxiety/ Depression/ Low mood/ Emotional support | 6 (25.0) |
| Medication | 6 (25.0) |
| Blood pressure check | 6 (25.0) |
| Cholesterol check | 2 (8.3) |
| Fatigue support | 1 (4.2) |
| Smoking cessation support | 1 (4.2) |
| Not-TIA related | 2 (8.3) |

**S15 Table: Feedback questionnaires from intervention participants (n=16)**

|  |  | n | (%) |
| --- | --- | --- | --- |
| **Checklist** |  |  |  |
| Checklist completed before appointment? | Yes | 3 | (18.8) |
|  | No | 13 | (81.3) |
|  | Missing | 0 | (0.0) |
| If yes, how easy was it to complete?* | Very easy | 7 | (53.8) |
|  | Quite easy | 5 | (38.5) |
|  | Neither | 1 | (7.7) |
|  | Quite difficult | 0 | (0.0) |
|  | Very difficult | 0 | (0.0) |
|  | Missing | 0 | (0.0) |
| Was the checklist used in the appointment? | Yes | 15 | (93.8) |
|  | No | 1 | (6.3) |
|  | Missing | 0 | (0.0) |
| **Appointment** |  |  |  |
| Were you able to discuss all your concerns or unmet needs in the appointment? | Yes | 16 | (100.0) |
|  | No | 0 | (0.0) |
|  | Missing | 0 | (0.0) |
| Did you feel the nurse/ therapist understood your concerns or needs? | Strongly agree | 11 | (68.8) |
|  | Agree | 4 | (25.0) |
|  | Uncertain | 1 | (6.3) |
|  | Disagree | 0 | (0.0) |
|  | Strongly disagree | 0 | (0.0) |
|  | Missing | 0 | (0.0) |
| Were you happy with the length of the appointment? | Yes | 16 | (100.0) |
|  | No- would have preferred it to be longer | 0 | (0.0) |
|  | No- would have preferred it to be shorter | 0 | (0.0) |
|  | Missing | 0 | (0.0) |
| Was the nurse able to address any of your concerns or needs during the appointment? | Yes | 16 | (100.0) |
|  | No | 0 | (0.0) |
|  | Missing | 0 | (0.0) |
| How satisfied were you with your appointment overall? | Very satisfied | 14 | (87.5) |
|  | Quite satisfied | 2 | (12.5) |
|  | Neither | 0 | (0.0) |
|  | Not satisfied | 0 | (0.0) |
|  | Not at all satisfied | 0 | (0.0) |
|  | Missing | 0 | (0.0) |
| **Action plan** |  |  |  |
| How satisfied are you with your action plan? | Very satisfied | 9 | (56.3) |
|  | Quite satisfied | 7 | (43.8) |
|  | Neither | 0 | (0.0) |
|  | Not satisfied | 0 | (0.0) |
|  | Not at all satisfied | 0 | (0.0) |
|  | Missing | 0 | (0.0) |
| Do you understand your action plan? | Strongly agree | 8 | (50.0) |
|  | Agree | 7 | (43.8) |
|  | Uncertain | 1 | (6.3) |
|  | Disagree | 0 | (0.0) |
|  | Strongly disagree | 0 | (0.0) |
|  | Missing | 0 | (0.0) |
| Do you feel confident that you will be able to complete your action plan? | Very confident | 7 | (43.8) |
|  | Quite confident | 7 | (43.8) |
|  | Neither | 1 | (6.3) |
|  | Not confident | 0 | (0.0) |
|  | Not at all confident | 0 | (0.0) |
|  | Missing | 1 | (6.3) |
| *N=13 |  |  |  |
