# Supplementary material for: Structured follow-up pathway to address unmet needs after transient ischaemic attack and minor stroke (SUPPORT TIA): Feasibility study and process evaluation
Source: PLoS One. 2025 Mar 13;20(3):e0317425. doi: 10.1371/journal.pone.0317425 (PMC11906085; doi:10.1371/journal.pone.0317425)
Supplement: Attachment — Submitted filename: SUPPORT TIA_Article Review_Sept022024.docx [file pone.0317425.s003.docx]

Dear Authors,

I have completed my review of your manuscript, *"Structured follow-up pathway to address unmet needs after transient ischemic attack and minor stroke (SUPPORT TIA): feasibility study and process evaluation”.* I commend the effort put into this important study, which addresses a critical aspect of post-stroke care.

**Overall Feedback:** Your research is timely and could significantly improve patient outcomes. However, I believe the manuscript could benefit from further clarification and enhancement in several areas:

**Suggestions for Improvement:**

1. **Introduction:**
   - Expand on the background to better highlight unmet needs in TIA and minor stroke follow-up care.
   - Clearly state the research gap and how your study addresses it.
2. **Methodology:**
   - Provide more details on participant selection criteria, including any exclusion criteria and how the sample size was determined.
   - Clarify the specific qualitative and quantitative methods used in the process evaluation and any validation techniques.
3. **Results:**
   - Offer a more detailed interpretation of the data, discussing potential confounding factors.
   - Consider adding figures to visually represent key data points.
4. **Discussion:**
   - Strengthen the connection between your findings and existing literature. Highlight how your results compare with or diverge from previous studies.
   - Emphasize the clinical implications of your findings and how the follow-up pathway could be implemented in practice.
5. **Conclusion:**
   - Clearly outline the next steps for research and offer concrete recommendations for healthcare providers based on your findings.

**Minor Revisions:**

- Ensure all abbreviations are defined upon first use.
- Improve the readability of the manuscript with thorough proofreading.

**Ethical Standards Compliance:** I noted that ethical approval was obtained from the relevant IRB, and informed consent was collected from all participants. This adherence to ethical standards is crucial and ensures respect for participants' rights.

**Adherence to Reporting Guidelines:** The manuscript follows relevant reporting guidelines, such as CONSORT, which enhances its clarity and transparency. The detailed account of study design, methodology, and results aligns with the expected standards for research reproducibility.

Thank you for the opportunity to review your valuable work. I believe these changes will strengthen your manuscript and increase its chances of publication. Please feel free to reach out if you need further clarification on my comments.

Thank you for the opportunity to review this valuable work.
