# Supplementary material for: Structured follow-up pathway to address unmet needs after transient ischaemic attack and minor stroke (SUPPORT TIA): Feasibility study and process evaluation
Source: PLoS One. 2025 Mar 13;20(3):e0317425. doi: 10.1371/journal.pone.0317425 (PMC11906085; doi:10.1371/journal.pone.0317425)
Supplement: Attachment — Submitted filename: Response to reviewers.docx [file pone.0317425.s005.docx]

Dear Editors,

Thank you for the opportunity to revise our manuscript. We have responded to the reviewer’s comments in a point-by-point format (in purple) detailed below.

I have completed my review of your manuscript, *"Structured follow-up pathway to address unmet needs after transient ischemic attack and minor stroke (SUPPORT TIA): feasibility study and process evaluation”.* I commend the effort put into this important study, which addresses a critical aspect of post-stroke care.

Thank you for the positive feedback.

**Overall Feedback:** Your research is timely and could significantly improve patient outcomes. However, I believe the manuscript could benefit from further clarification and enhancement in several areas:

**Suggestions for Improvement:**

1. **Introduction:**
   - Expand on the background to better highlight unmet needs in TIA and minor stroke follow-up care.

We have added further detail to highlight unmet needs:

Page 4, lines 9-11: *“Qualitative research indicates information is difficult to process at the time of diagnosis; language was too medical; clinicians gave contradictory advice; and information was too generic and not personalised.”*

Page 4, lines 14-15: *“This is further exacerbated by, loss of confidence, changes in family roles and dynamics and driving restrictions.”*

Page 4, lines 17-18: *“Clinicians commonly lack awareness of residual problems and holistic needs after TIA and minor stroke.”*

- Clearly state the research gap and how your study addresses it.

We have added a sentence to clarify the research gap (page 4, lines 19-21)

*“**Care needs after TIA and minor stroke include information provision (diagnosis and stroke risk); stroke prevention (medication and lifestyle change); and holistic care (residual problems and return to work or usual activities).”*

This precedes the sentence which describes how our study aims to address this (*“We therefore developed a multifaceted intervention to identify and address unmet needs after TIA and minor stroke…”*).

2. **Methodology:**
   - Provide more details on participant selection criteria, including any exclusion criteria and how the sample size was determined.

We have added additional detail of the inclusion criteria. The full eligibility criteria are published in our protocol paper.

Page 5, lines 16-17: *“… had ability to converse in everyday English and read in English and capacity to provide fully informed consent”*

Justification for how the sample size was determined is reported on page 11, lines 4-7:

*“As this was a feasibility study, no formal sample size calculation was performed; however, the sample size was the estimated number that would demonstrate the potential for sufficient recruitment rate of our target population.(24)”*

- Clarify the specific qualitative and quantitative methods used in the process evaluation and any validation techniques.

Table 1 summarises methods and outcomes corresponding to the process evaluation objectives. Structured observations, feedback questionnaires and qualitative methods are further described in the text (page 9). Given that our protocol, with detailed methodology, has been published, we have presented a summary of methods for brevity. Validation techniques were not used.

3. **Results:**
   - Offer a more detailed interpretation of the data, discussing potential confounding factors.

Interpretation of the data has been presented in the discussion (page x, subheading “*Interpretation of findings and comparison with literature*”).

As this is a feasibility study, we have presented aggregate data for patient reported outcomes (Table 4) and do not have adequate sample size to explore confounding factors. Randomisation used a minimisation algorithm with the following variables: age at consent (<60 years, ≥60 years); sex (male, female); diagnosis (TIA, minor stroke); employment (employed, non-employed/retired) to ensure that these potential confounding factors were equally distributed between groups. We have added a sentence to the randomisation section in the methods to clarify this;

Page 7, lines 3-4: “*To ensure that these potential confounding factors were equally distributed between groups, a minimisation algorithm was used with the following variables…”*

- Consider adding figures to visually represent key data points.

Thank you for this suggestion, we have added Figure 4 (page 24) to summarise findings from the intervention process evaluation.

4. **Discussion:**
   - Strengthen the connection between your findings and existing literature. Highlight how your results compare with or diverge from previous studies.

We have added further comparison to existing literature (page 36, lines 5-9):

*“There are few interventions focused on care after TIA and minor stroke that address holistic needs. Scoping reviews demonstrate that previous research focused largely on secondary prevention and management of increased risk of further stroke.[REFs] Similar to our intervention, most studies delivered the intervention in an outpatient clinic setting and used low-tech and low-cost materials such as exercise booklets and diaries.”*

- Emphasize the clinical implications of your findings and how the follow-up pathway could be implemented in practice.

We have added a paragraph discussing implementation (page 38, lines 14-19):

*“There is variation in terms of TIA clinic care pathways in the UK.(7) Therefore, implementation requirements for our follow-up pathway would be context specific. For example, where a nurse-led follow-up is already in place, implementation may only require restructuring to incorporate intervention components such as the checklist and resources. However, for services with no nurse follow-up, implementation would require commissioning. Further research is required to explore barriers and enablers to implementation in different TIA clinic settings.”*

5. **Conclusion:**
   - Clearly outline the next steps for research and offer concrete recommendations for healthcare providers based on your findings.

We have highlighted next steps for research: *“Future research should move to a full-scale definitive trial and health economics evaluation to evaluate the effectiveness and cost-effectiveness of the intervention.”* (page 39, lines 19-21)

We have added a sentence to provide recommendations for healthcare providers:

“*Healthcare providers should consider incorporating a checklist, which encompasses information provision, stroke prevention and holistic care, to improve communication and identification of needs.*” (page 39, lines 17-19)

**Minor Revisions:**

- Ensure all abbreviations are defined upon first use.

All abbreviations have now been defined upon first use.

- Improve the readability of the manuscript with thorough proofreading.

The manuscript has been proofread.

**Ethical Standards Compliance:** I noted that ethical approval was obtained from the relevant IRB, and informed consent was collected from all participants. This adherence to ethical standards is crucial and ensures respect for participants' rights.

No edits required.

**Adherence to Reporting Guidelines:** The manuscript follows relevant reporting guidelines, such as CONSORT, which enhances its clarity and transparency. The detailed account of study design, methodology, and results aligns with the expected standards for research reproducibility.

No edits required.

Thank you for the opportunity to review your valuable work. I believe these changes will strengthen your manuscript and increase its chances of publication. Please feel free to reach out if you need further clarification on my comments.

Thank you for the opportunity to review this valuable work.

Thank you for your kind words.

Best wishes,
Dr Grace Turner

University of Birmingham
